# Substitutions in SurA and BamA Lead to Reduced Susceptibility to Broad Range Antibiotics in Gonococci

**DOI:** 10.3390/genes12091312

**Published:** 2021-08-25

**Authors:** Ivan Bodoev, Maja Malakhova, Julia Bespyatykh, Dmitry Bespiatykh, Georgij Arapidi, Olga Pobeguts, Victor Zgoda, Egor Shitikov, Elena Ilina

**Affiliations:** 1 Federal Research and Clinical Center of Physical-Chemical Medicine, 119435 Moscow, Russia; maja_m@mail.ru (M.M.); juliabespyatykh@gmail.com (J.B.); d.bespiatykh@gmail.com (D.B.); arapidi@gmail.com (G.A.); nikitishena@mail.ru (O.P.); egorshtkv@gmail.com (E.S.); ilinaen@gmail.com (E.I.); 2 Moscow Institute of Physics and Technology, State University, 141701 Dolgoprudny, Russia; 3 Orekhovich Institute of Biomedical Chemistry, Russian Academy of Medical Sciences, 119121 Moscow, Russia; victor.zgoda@gmail.com

**Keywords:** *Neisseria gonorrhoeae*, *bamA*, *surA*, multidrug resistance, efflux pumps

## Abstract

There is growing concern about the emergence and spread of multidrug-resistant *Neisseria gonorrhoeae.* To effectively control antibiotic-resistant bacterial pathogens, it is necessary to develop new antimicrobials and to understand the resistance mechanisms to existing antibiotics. In this study, we discovered the unexpected onset of drug resistance in *N. gonorrhoeae* caused by amino acid substitutions in the periplasmic chaperone SurA and the β-barrel assembly machinery component BamA. Here, we investigated the i19.05 clinical isolate with mutations in corresponding genes along with reduced susceptibility to penicillin, tetracycline, and azithromycin. The mutant strain NG05 (*surA^mut^ bamA^mut^*, and *penA^mut^*) was obtained using the pan-susceptible n01.08 clinical isolate as a recipient in the transformation procedure. Comparative proteomic analysis of NG05 and n01.08 strains revealed significantly increased levels of other chaperones, Skp and FkpA, and some transport proteins. Efflux pump inhibition experiments demonstrated that the reduction in sensitivity was achieved due to the activity of efflux pumps. We hypothesize that the described mutations in the *surA* and *bamA* genes cause the qualitative and quantitative changes of periplasmic chaperones, which in turn alters the function of synthesized cell envelope proteins.

## 1. Introduction

*Neisseria gonorrhoeae* is the causative agent of gonorrhea, one of the most common sexually transmitted diseases. An estimated 78 million people are infected with this bacterium annually worldwide [1]. The rate of gonorrhea infections is higher among men; however, infections with serious complications are more prevalent in women, since urogenital infections in women are often asymptomatic. If gonorrhea is left untreated, the additional morbidity may be severe and include pelvic inflammatory disease, serious eye infections in newborns, problems with the reproductive system, including infertility, and enhanced transmission of HIV in both women and men [2].

Since the beginning of the antimicrobial era, *N. gonorrhoeae* has repeatedly demonstrated its extraordinary ability to develop resistance to all antimicrobial agents administered to treat the infection [3]. Following the introduction of a new drug, resistance spread worldwide in only one to two decades. Although resistance to dual therapy (250 mg ceftriaxone with 1 g azithromycin), currently recommended by the World Health Organization, is rare, *N. gonorrhoeae* strains with reduced sensitivity to extended-spectrum cephalosporins and resistance to azithromycin have been reported, raising concerns that the effectiveness of this treatment will be short-lived [4,5].

Gonococci use a wide variety of known antimicrobial resistance (AMR) mechanisms: inactivation of drugs, alteration of antimicrobial targets, increased efflux and decreased uptake [6,7]. Such diversity is associated with specific chromosomal mutations and with the natural competence of the gonococcus, which allows the uptake and incorporation of both plasmid and chromosomal DNA from related and divergent species [7,8]. Of particular concern are non-specific mechanisms of drug resistance that alter the permeability of gonococcal cells or increase the efflux. These mechanisms can affect a wide range of antimicrobial agents with various modes of action, including penicillins, cephalosporins, tetracyclines, and macrolides. A striking example is a modification of porins, the outer membrane proteins (OMPs), which leads to the decreased diffusion of antibiotics into the periplasmic space. For instance, the resistance of *N. gonorrhoeae* to сephalosporin may be caused by the mutations in the porin coding gene *porB*, resulting in amino acid substitution in loop 3 of the PorB1b protein [9]. The development of penicillin resistance in gonococcus is also contributed by the activation of efflux cell systems, primarily, the MtrCDE system. Mutations in the *mtrR* gene that encodes the transcriptional repressor of the MtrCDE system and in its promoter were shown to elevate the penicillin minimal inhibitory concentration (MIC) up to 0.25–0.5 mg/L [10,11].

To date, the molecular mechanisms of antibiotic resistance in gonococcus have been described quite extensively [3,12]. However, new mutations responsible for resistance are still being discovered. Examples of recently found variations are a 6-bp deletion in the *bla*_TEM-1_ gene and an atypical A2059G mutation in the 23S rRNA gene resulting in resistance to penicillin and azithromycin, respectively [13,14]. Nevertheless, some strains are still found, with a phenotype that cannot be explained by the presence of known resistance markers [15,16]. In particular, the i19.05 *N. gonorrhoeae* clinical isolate was drawn from a laboratory strain collection tested earlier [17]. The strain was characterized by a reduced susceptibility to penicillin (MIC = 0.5 mg/L), tetracycline (MIC = 0.5 mg/L), and azithromycin (MIC = 1.0 mg/L). Interestingly, this strain does not contain known genetic resistance determinants except for the penicillin resistance determinant PenA Asp345A [18], which cannot explain the resistant phenotype. In addition, our attention was drawn to the presence of a previously unreported amino acid substitution in SurA, and several common substitutions in BamA. According to recent reports, alterations leading to the absence of the periplasmic chaperones SurA and Skp affect bacterial resistance to antibiotics in *Pseudomonas aeruginosa* and *Salmonella typhimurium*, respectively [19,20]. SurA and Skp are chaperones that transfer newly synthesized proteins of the outer membrane through the periplasm to the β-barrel assembly machinery (BAM). BamA is the main protein of the BAM system, which is involved in the assembly and insertion of β-barrel proteins (porins and efflux channels) into the outer membrane [21,22,23].

In the present study, we investigated the impact of substitutions in SurA and BamA on increasing levels of drug resistance. For this, a pan-susceptible n01.08 recipient strain was consequently transformed by *penA^mut^*, *bamA^mut^*, and *surA^mut^* genes amplified from the i19.05 genome. Comparative proteomic analysis of donor, recipient, and transformant strains followed by experiments on the inhibition of efflux pumps allowed us to assume the existence of a new mechanism of drug resistance formation in gonococci due to efflux pump activation determined by mutant forms of periplasmic SurA and BamA.

## 2. Materials and Methods

### 2.1. Bacterial Strains and Culture Conditions

The i19.05 and n01.08 *N. gonorrhoeae* clinical isolates [17] were used in this study. Gonococcal strains were cultured from frozen stocks (−80 °C) onto the BBL GC agar base (Becton Dickinson & Co, Franklin Lakes, NJ, USA) supplemented with 1% BBL IsoVitaleX Enrichment (Becton Dickinson & Co, Franklin Lakes, NJ, USA) at 37 °C in a 5% CO_2_ enriched atmosphere for 20–24 h. For proteomic analysis, strains were cultured on Chocolate Agar (BIOMERIEUX, Marcy-l’Étoile, France) supplemented with 1% BBL IsoVitaleX Enrichment in the same conditions for 18 h with and without additionally added ampicillin (0.12 mg/L).

Susceptibility testing to penicillin G, ceftriaxone, tetracycline, ciprofloxacin, spectinomycin, and azithromycin (all from MilliporeSigma, Burlington, MA, USA) was performed by the agar dilution method according to CLSI recommendations. *N. gonorrhoeae* strain ATCC 49226 was used as a control. Current CLSI interpretive criteria were used to define antimicrobial resistance (https://clsi.org/standards/products/microbiology/documents/m100/ accessed on 23 March 2021).

### 2.2. DNA Extraction

Total genomic DNA from tested *N. gonorrhoeae* strains was isolated by a “DNA express” kit (Lytech Ltd., Moscow, Russia). If necessary, the prepared DNA samples were stored at a temperature of −20 °C.

For whole genome sequencing (WGS), bacterial lysis was carried out using the Promega Nuclei buffer (Promega, Madison, WI, USA). A saturated NaCl solution was added to remove cellular proteins. DNA was concentrated and desalted by isopropanol deposition. TE buffer (50–100 µL) was added to the DNA precipitate for further storage at 4 °C. DNA was additionally purified using mini-columns for DNA purification (Technoclone, Moscow, Russia).

### 2.3. Convenient PCR and Sanger Sequencing

Genomic DNA amplification and sequencing were done as previously described [15]. Oligonucleotide primers are listed in Supplementary Table S4.

### 2.4. Spot-Transformation

The NG01 (*penA^mut^*) and NG05 (*penA^mut^, bamA^mut^,* and *surA^mut^*) strains were constructed during the consequent transformation of the pan-susceptible n01.08 (recipient) strain by DNA fragments containing *penA^mut^*, *bamA^mut^*, and *surA^mut^* genes from i19.05. Spot-transformation of gonococci was performed as described earlier [15].

The 4377 bp, 5399 bp, and 6758 bp fragments of *N. gonorrhoeae* chromosomal DNA, which included corresponding sequences of *penA*, *bamA,* and *surA* genes as well as the nearest DNA uptake sequences (DUS12, 5′ ATGCCGTCTGAA 3′) on their ends, were amplified with primers presented in Supplementary Table S4. Amplification was done using the i19.05 (mutant) genomic DNA as a template.

Each DNA fragment contained mutant variants of analyzed genes that were used individually for the transformation of the n01.08 recipient strain. The transformants were selected on the GC agar plates supplemented with ampicillin.

### 2.5. Whole Genome Sequencing

DNA (300 ng for each sample) was fragmented using the Covaris S220 system (Covaris, Woburn, MA, USA) up to a final size of 300–500 bp according to the manufacturer’s recommendations. The DNA libraries of n01.08 and NG05 were prepared with the Ion Xpress Plus Fragment Library Kit (Thermo Fisher Scientific, Waltham, MA, USA) for sequencing on the Ion Torrent PGM (Thermo Fisher Scientific, Waltham, MA, USA). The Ion PGM Template OT2 200 Kit (Thermo Fisher Scientific, Waltham, MA, USA) was used for emulsion PCR. DNA sequencing was performed using Ion 318 chip v2 and the Ion PGM Sequencing 200 Kit v2 (Thermo Fisher Scientific, Waltham, MA, USA).

Previously, WGS of *N. gonorrhoeae* clinical isolate i19.05 was done on a Roche Genome Sequencer GS FLX (Roche Holding, Basel, Switzerland) using a standard protocol for a shotgun genomic library. The accuracy of raw reads was improved with spectral alignment error correction tool SAET 3 and the Newbler v.2.6 (Roche Holding, Basel, Switzerland) was used for assembly.

The sequencing results were deposited in the NCBI database under PRJNA236643 and PRJNA243883 accession numbers. Accession numbers of assembled strains deposited to the NCBI Assembly database are GCA_000695425.1-i19.01, GCA_000705675.1-n01.08, and GCA_000763255.1-NG05.

### 2.6. SNP Analysis

Raw reads were mapped to the *N. gonorrhoeae* FA1090 (AE004969.1) reference sequence with Bowtie2 [24]. SNP calling was done using the samtools mpileup tool [25]. SnpEff was used for SNP annotation and effect prediction [26]. Genes of interest and typing genes were manually curated. The BLAST web server (https://blast.ncbi.nlm.nih.gov/Blast.cgi accessed on 6 April 2021) was used to compare gene sequences with those available in the NCBI database. The domain structure of the protein was determined using MPI Bioinformatics toolkit and InterPro [27,28].

### 2.7. Protein Extraction and Trypsin Digestion

For proteomic analysis, the bacterial cells were harvested from Chocolate Agar supplemented with 1% BBL IsoVitaleX Enrichment into an ice-cold TE buffer (pH 8.1), storing this bacterial suspension for at least 30 min at −80 °C. Cells were disrupted by 5 min ultrasonic disintegration in a Branson Sonifier 250 (Branson Ultrasonics, Brookfield, CT, USA) (duty cycle: 40%, output control: 7). Unbroken cells were removed by centrifugation (12,000× *g*, 15 min, 4 °C). Cell lysates were fractionated on a cell envelope (CE) (including the outer membrane, the periplasmic, and the inner membrane) and cytosol (C) by ultracentrifugation for 8 min at 170,000× *g*. The soluble cytosol fraction was collected, and the resulting pellet with a cell envelope fraction was dissolved in 1 mL of 2 mM Tris-HCl, pH 7.6, and used for further analysis. Protein pools for NG05 and i19.05 strains cultivated on media with 0.12 mg/L ampicillin were also obtained.

Protein concentration was measured by the Bradford method [29] using the Bradford Protein Assay Kit (Bio-Rad, Hercules, CA, USA). Proteolytic in-gel digestion was performed as described previously [30].

### 2.8. LC-MS/MS Analysis

To identify differences in the abundance of other proteins in the cell, a comprehensive proteomic analysis via LC-MS/MS was performed on a Q-Exactive HF hybrid quadrupole-Orbitrap mass spectrometer (Thermo Fisher Scientific, Waltham, MA, USA) with a nanoelectrospray source Nanospray Flex (Thermo Fisher Scientific, Waltham, MA, USA) coupled to an Ultimate 3000 RSLCnano (Dionex, Sunnyvale, CA, USA) chromatography system. Liquid chromatographic separation was performed on a reverse-phase column 15 cm × 75 μm i.d. (Agilent Technologies, Santa Clara, CA, USA) packed with Zorbax 300SB-C18 resin (particle size–3 µm, pore diameter–100 A). The HPLC system was configured in a trap-elute mode. Samples were loaded on a 2 cm × 75 μm i.d. Acclaimed PepMap trap column (Dionex, Sunnyvale, CA, USA), with C18 resin with 3 um particles with 100 A pores, with a 2 µL/min flow of Solvent A (0.1% *v*/*v* formic acid) for 5 min. Peptides were eluted with a gradient of 5–40% (*v*/*v*) of Solvent B (0.1% *v*/*v* formic acid, 79.9% *v*/*v* acetonitrile) over 120 min at a flow rate of 300 nL/min. After each elution system and columns were washed with 99% of Solvent B for 10 min and regenerated with 5% of Solvent B for 10 min.

The mass spectrometer was operated in a positive mode in a data-dependent experiment with survey scans acquired at a resolution of 700 at *m/z* 400 within an *m/z* range of 400–1500 and with automatic gain control set for 106 and a maximum injection time of 50 ms. As many as 20 of the most abundant precursor ions with a charge of +2 and above from the survey scan were selected for HCD fragmentation. The normalized collision energy was 30. MS2 spectra were acquired at a resolution of 17,500 at *m*/*z* 400, automatic gain control was set to 105, and the maximum injection time was set to 100 ms. After fragmentation, ions were dynamically excluded from consideration for 10 s with a 5 ppm window.

### 2.9. Peptide Synthesis

The isotopically labeled standard samples of target peptides were obtained using the solid-phase peptide synthesis on the Overture (Gyros Protein Technologies AB, Tucson, AZ, USA) according to the published method [31]. Isotope-labeled lysine (^13^C_6_,^15^N_2_) or arginine (^13^C_6_,^15^N_4_) was used for isotope-labeled peptide synthesis instead of the unlabeled lysine. The concentration of the synthesized peptides was quantified through acidic hydrolysis, followed by the analysis of derived amino acids with fluorimetric detection [32].

### 2.10. Liquid Chromatography-Selected Reaction Monitoring Analysis

The liquid chromatography-selected reaction monitoring (LC-SRM) approach was carried out for the proteins isolated from whole-cell lysates for i19.05, NG05, and n01.08 strains. To identify the effects of antibiotics, the cells were cultured on media with and without ampicillin. Reconstituted peptides were separated using an Infinity 1290 UPLC system (Agilent Technologies, Santa Clara, CA, USA) comprising an autosampler thermostat with an installed 20-µL-loop column thermostat equipped with a 6-port valve and a binary pump. Twenty microliters of samples were loaded onto an Eclipse C18 (2.1 × 50 mm, 1.8 µm particles size) column (Agilent Technologies, Santa Clara, CA, USA). This column was constantly heated at 45 °C. A linear gradient of Mobile Phase A (water, pH 2.85) and Mobile Phase B (acetonitrile) were both supplied with 0.09% formic acid and 0.01% trifluoroacetic acid. The gradient started from 1.5% of B and isocratically lasted for 1.5 min following an increase in the content of B to 33% for the next 34.5 min at a 0.3 mL/min flow rate. The column was washed in 85% of B for 4 min at 0.4 mL/min and equilibrated in the initial gradient conditions (1.5% of B) for 6 min at 0.3 mL/min before the next run.

Peptides were detected on a triple-quadrupole G6495 mass spectrometer (Agilent Technologies, Santa Clara, CA, USA) in the dynamic selected reaction monitoring mode. The instrument was operated in positive electrostatic ionization mode and was equipped with a Jet-Stream ionization source. Drying gas (nitrogen) temperature was set to 250 °C, and the flow rate was adjusted to 14 L/min, whereas the sheath gas (nitrogen) temperature was 280 °C, and the flow rate was 11 L/min. The capillary voltage was 3.7 kV and the nozzle voltage was specified to 450 V. Both precursor and fragment ions were isolated by the first and third quadrupole, respectively, in a narrow ±0.65 u (Unit mode) isolation window and within a retention time scheduled in a ±0.4 min detection window. Collision energy and cell accelerating voltage were optimized for each transition during peptide selection and detection method development. The complete duty cycle was estimated to be 1600 ms with a dynamic dwell time depending on the number of concurrent transitions.

### 2.11. Protein Identification and Quantitation

Raw data were captured from the mass spectrometer and converted to Mascot Generic Format files using ProteoWizard with the following parameters: peakPicking true 2, msLevel 2, and zeroSamples removeExtra. For thorough protein identification and quantification, raw LC-MS/MS data were analyzed with MaxQuant v. 1.6.10.43 against the UniProt knowledgebase, i.e., the taxon *N. gonorrhoeae* (strain ATCC 700825/FA1090). For this procedure, the following parameters were used: an Orbitrap instrument type, tryptic digestion with two possible missed cleavages, fixed modification for carbamidomethyl (C), variable modifications for oxidation (M), acetyl (protein N-term), and LFQ (label-free quantification). A 1% false discovery rate threshold was applied to search results from individual datasets. Frequently observed contaminants, such as trypsin, bovine proteins, and human keratins, were removed from the results. The mass spectrometry proteomics data have been deposited to the ProteomeXchange Consortium (http://proteomecentral.proteomexchange.org) via the PRIDE partner repository with the dataset identifier PXD022993.

Data obtained after LC-SRM analysis were processed using Skyline (64-bit v. 3.7.0.113117) software. The target protein library was built from a custom FASTA file. The target peptide list was built via in silico digestion with trypsin, and any missed cleavage was forbidden. Transitions were calculated as monoisotopic masses, and the prediction of retention time was applied based on the custom calculated values. The peptides were represented in native and in stable isotope-labeled forms with the incorporation of C-terminal residues of [^13^C_6_^15^N_4_]-Arg or [^13^C_6_^15^N_2_]-Lys. Statistics were performed using GraphPad Prism 7.04 software, as described for each experiment in the table or figure legends.

The Clusters of Orthologous Groups (COG) were annotated by aligning the amino acid sequences of *N. gonorrhoeae* genes to the COG-NCBI database (https://www.ncbi.nlm.nih.gov/research/cog-project/ accessed on 6 April 2021) using the DIAMOND aligner (v. 2.0.9). Gene Ontology (GO) terms, UniProt (UP) keywords, and KEGG (Kyoto Encyclopedia of Genes and Genomes) pathway enrichment analysis was performed using the DAVID database, and a *p*-value of <0.05 was considered to indicate significant enrichment. In this analysis, we filtered the significantly differentially expressed genes (DEGs) with a fold change (FC) of ≥1.25 or ≤0.8 and with *p*-values of <0.05. Quantitative proteome analysis was performed for both fractions of pairs: (1) NG05 vs. n01.08, (2) NG05 MA vs. n01.08, (3) i19.05 vs. n01.08, (4) i19.05 MA vs. n01.08, (5) i19.05 MA vs. i19.05, and (6) NG05 MA vs. NG05. In total, we identified protein pools with abundances that were significantly different between tested groups (*p* < 0.05, FC > 1.25) (Supplementary Table S2).

### 2.12. Ethidium Bromide Whole-Cell Accumulation Assays

Strain cultures in the exponential growth phase were harvested and diluted to OD600 nm = 0.5 in Hanks’ Balanced Salt solution. In a 96-well plate format (Grainer Bio-one), basic fluorescence (emission: 320 nm/extinction: 612 nm) was measured every 5 min. Ethidium bromide at a concentration of 10 µg/mL was subsequently added to each sample. Phe-Arg-β-Naphthylamide (PaβN) (MilliporeSigma, Burlington, MA, USA) at 50 µg/mL was used as an efflux pump inhibitor (EPI) to control for the role of efflux pumps in ethidium-bromide-mediated fluorescence [33]. Measurements were performed in triplicate, and the kinetics of ethidium bromide uptake were monitored from 2 to 45 min at 37 °C in a microplate reader (Fluoroskan Ascent, Thermo Fisher Scientific, Waltham, MA, USA). Results were normalized against the autofluorescence of ethidium bromide and are depicted relative to the total amount of protein.

The influence of PAβN on the drug’s MIC was performed by the agar dilution method as described above. Gonococcal strains were cultured onto the BBL GC agar base supplemented with 50 µg/mL PAβN and ampicillin (from 0.02 to 0.5 µg/mL). Plates without PAβN were used as a control.

## 3. Results

### 3.1. Genetic Characteristics of N. Gonorrhoeae Strains

This study focused on the characterization of the *N. gonorrhoeae* clinical isolate i19.05 with reduced susceptibility to penicillin (MIC = 0.5 mg/L), tetracycline (MIC = 0.5 mg/L), and azithromycin (MIC = 1.0 mg/L). The strain carried four missense mutations in the *surA* (NGO1714) gene and two nsSNPs in *bamA* (NGO1801) relative to the reference drug-susceptible strain FA1090 and had no drug resistance markers other than PenA Asp345A. According to BLAST analysis, SurA substitutions Val61Ala, Lys206Glu, and Ala230Thr are often found among members of the genus, while Asn105Ser was unique for the i19.05 *N. gonorrhoeae* isolate [34]. Of these, Val61Ala and Asn105Ser substitution were found in the N-domain, the Lys206Glu and Ala230Thr changes were in the parvulin-like peptidylprolyl isomerase domain. In addition, two substitutions in BamA (Leu568Trp and Ser585Leu) were present in other *Neisseria spp*. strains represented in the NCBI database (Supplementary Table S1). Both substitutions in BamA were found in the transmembrane β-barrel domain.

For transformation experiments, a naturally competent, pan-susceptible *N. gonorrhoeae* n01.08 strain was used. The strain carried two substitutions in the SurA protein, Val61Ala and Lys206Glu, which were identical to i19.05. Several substitutions were observed in BamA: Phe116Leu, Ala282Val, Arg553Lys, Lys554Gln, and Ser585Leu (Supplementary Table S1).

### 3.2. Generation of N. gonorrhoeae Mutant Strains

Showing that the mutant variants of SurA and BamA proteins can affect a drug-resistant phenotype in the i19.05 strain, two *N. gonorrhoeae* strains were constructed: NG01 (*penA^mut^*) and NG05 (*penA^mut^, bamA^mut^,* and *surA^mut^*). To evaluate changes for all strains used in this study, the ampicillin, tetracycline, and clarithromycin MICs were measured. The MICs of NG05 for all antibiotics were increased relative to n01.08, while NG01 (*penA^mut^*) showed increased resistance to ampicillin only (Table 1).

### 3.3. Proteome Analysis of N. gonorrhoeae

**Figure 1:**
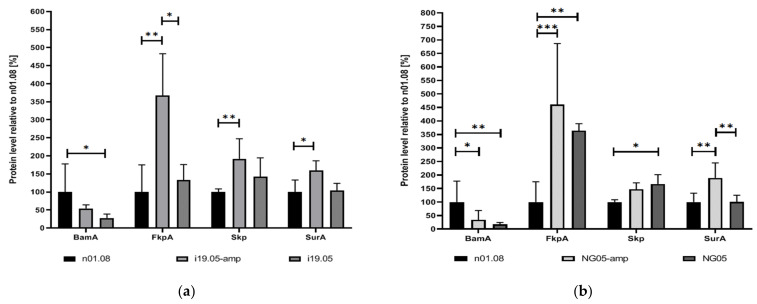
Quantification of mass spectrometry analysis for BamA, SurA, Skp, and FkpA proteins. Comparison of protein levels between n01.08 and mutant strains i19.05 (**a**) and NG05 (**b**). Asterisks indicate significant differences (* *p* < 0.05, ** *p* < 0.01, *** *p* < 0.001) compared to n01.08 using ANOVA analysis. Amp, Ampicillin.

To determine the possible effect of mutations on the quantitative differences of proteins in the cell, the LC-SRM approach was carried out for the proteins encoded by the mutant genes as well as for other periplasmic chaperones: Skp (OmpH family outer membrane protein; NGO1802) and FkpA (Peptidyl-prolyl cis-trans isomerase; NGO1225). SRM analysis showed an increase in the amount of SurA protein in the mutant strains i19.05 and NG05 when cultivated on media supplemented with ampicillin (Figure 1). The overabundance of two other periplasmic chaperones, Skp and FkpA, relative to the recipient strain was observed in both strains on media with and without an antibiotic. On the contrary, the BamA level was reduced in the i19.05 and NG05 strains, irrespective of the cultivation medium.

A comprehensive proteomic analysis via LC-MS/MS revealed a total of 1125 proteins in the CE fraction, 894 of which were common to all tested strains (i19.05, n01.08, and NG05). Proteomics of the C fraction in the same experiment yielded 928 proteins, 676 of which were shared among all strains. Proteome coverage for both fractions ranged from 52.72% (1111 proteins) in n01.08 to 54.53% (1149 proteins) in i19.05 (Supplementary Table S2).

We found 275 differentially abundant proteins (DAPs) in NG05 strains (FC ≥ 1.25 or ≤ 0.8 and *p*-values < 0.05) when compared to the proteome profile of n01.08: 110 and 165 in the CE and C fractions, respectively. To better understand the functions and relationships of the differentially abundant proteins, we performed COG functional classification for both fractions (Supplementary Figure S1). The highest scoring categories in the CE fraction were “extracellular structures” and “cell motility,” which included 23.5% and 17.4% upregulated proteins of the total in the categories, respectively. Many of the DAPs were involved in “lipid transport and metabolism,” “carbohydrate transport and metabolism,” “amino acid transport and metabolism,” “coenzyme transport and metabolism,” and “inorganic ion transport and metabolism”.

In the C fraction, the majority of DAPs were predicted to be involved in “cell cycle control, cell division, [and] chromosome partitioning” (24%), “post-translational modification, protein turnover, and chaperones” (14.6%), and “intracellular trafficking, secretion, and vesicular transport” (13.3%). In addition, “extracellular structures” (11%) and “cell motility” (13%) categories were enriched with proteins, as in the CE fraction. The enrichment of the categories “extracellular structures” and “cell motility” was associated with the ubiquitous abundance of Pil proteins in both fractions.

Functional enrichment analysis was performed using the DAVID database to determine the properties of all 275 DAPs (Supplementary Table S3). The results for the biological process categories indicated that the upregulated proteins were related to translation (GO:0006412). In terms of molecular function, the upregulated DAPs were enriched in structural constituents of the ribosome (GO:0003735), manganese ion binding (GO:0030145), and iron-sulfur cluster binding (GO:0051536). For the analysis of cellular components, the upregulated DAPs were mainly located in the ribosome (GO:0005840). Furthermore, the KEGG pathway enrichment analysis revealed the association of the DAPs with genetic information processing “Ribosome” (ngo03010). In addition, the overlapping UniProtKB Keywords included: “Manganese,” “Ribonucleoprotein,” “Ribosomal protein,” “rRNA-binding,” and “Ribosome biogenesis”.

Under antibiotic treatment, the proteome of NG05 had more differences relative to n01.08. Comparative analysis of two strains revealed 216 and 189 DAPs in the CE and C fractions, respectively. Of these, components of the MacAB-TolC efflux pump system were remarkably increased. Proteomic profiling revealed that the amount of MacA, MacB, and MtrE (TolC homolog) was increased in 3.14-, 1.94-, and 1.23-fold (*p* < 0.05), respectively. It is important to note that in the initial i19.05 strain relative to n01.08, the abundance of MacA protein was increased five-fold without ampicillin and eleven-fold with ampicillin (*p* < 0.001). Also, in i19.05 the components of the MtrCDE efflux system were constantly overabundant in comparison with n01.08. Changes in the abundance of porins in the studied samples were unreliable and could not indicate the presence of this resistance mechanism.

### 3.4. PAβN Reduces the Intrinsic Resistance of N. Gonorrhoeae to Antibiotics

**Figure 2:**
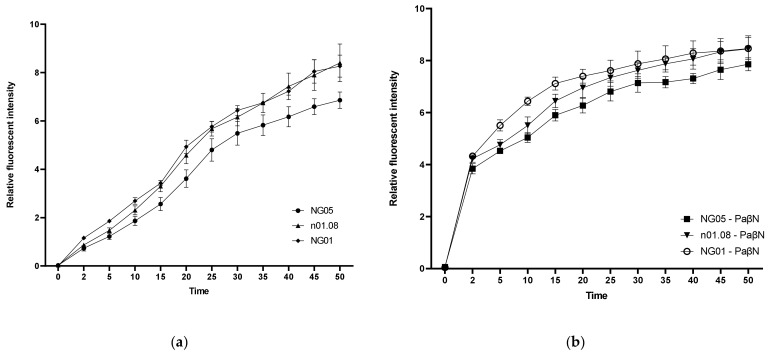
Fluorescence emission of ethidium bromide from *N. gonorrhoeae* strains. Efflux level of n01.08, NG01, and NG05 (**a**) and with the addition of PaβN as an EPI (**b**). Data depict the mean and SD of at least 3 experiments. ANOVA analyses revealed significant differences (*p* < 0.0136) for both n01.08 vs. NG05 and NG01 vs. NG05 in the time range between 35 and 45 min.

To determine the efflux activity of mutant strains, ethidium bromide whole-cell accumulation assay measurements with and without an EPI were carried out. It was found that NG05 took up ethidium bromide significantly less efficiently than n01.08 and NG01 after 15 min and reached lower values (Figure 2A). The level of ethidium bromide uptake in all strains upon the inhibition of efflux processes by PAβN was higher. The total increase in relative fluorescent intensity after 45 min in NG05 with an EPI was lower, but the difference was not significant (*p* > 0.24) in comparison with n01.08 and NG01 (Figure 2B).

To assess the effect of efflux on the level of bacterial resistance to antibiotics, MICs of ampicillin, tetracycline, and clarithromycin were determined for n01.08, NG01, and NG05 in the presence and absence of PAβN (Table 1). Despite the difference in the resistance of NG05 and n01.08 strains after the addition of EPI, the MIC values of clarithromycin and tetracycline become 0.19 and 0.125 mg/L, respectively, for both strains. In the case of ampicillin, the addition of an EPI to the studied strains decreased the MIC values twofold. However, the MIC for the NG05 strain was 0.012 mg/L, which is still higher than 0.03 mg/L for the n01.08 strain.

## 4. Discussion

To our knowledge, this is the first study focused on the role of mutations in *surA* and *bamA* genes in reducing gonococcal antibiotic susceptibility. Recently, it was demonstrated that the products of these genes directly interact and mediate the β-barrel OMP biogenesis, while the altered forms of the proteins may affect the AMR profile [35]. The investigated strain i19.05 carried four substitutions in SurA relative to the reference FA1090 strain, only one of which has not been previously described (Supplementary Table S1). We also found several substitutions in BamA; however, they were common for *Neisseria* species. Since i19.05 showed reduced sensitivity to different classes of antibiotics, we assumed that such a composition of amino acid sequences of SurA and BamA may lead to qualitative and quantitative changes of the OMPs and therefore mediate antibacterial resistance.

To support this suggestion, we obtained the NG05 (*penA^mut^, bamA^mut^*, and *surA^mut^*) strain with reduced sensitivity to ampicillin, tetracycline, and clarithromycin and the NG01 (*penA^mut^*) strain demonstrating reduced sensitivity to ampicillin only, thereby showing that a mutation in the *penA* gene cannot lead to such a broad resistance phenotype. Multiple attempts to construct a transformant carrying the isolated *surA^mut^* gene were unsuccessful. The possible explanation is that, for the correct functioning of mutant SurA, it should directly interact with the appropriate variant of the BamA protein, which was different in the recipient strain (Supplementary Table S1).

A comparative proteomic analysis by mass spectrometry was employed to determine the effect of substitutions on the protein profile since this method is widely used to study the mechanisms underlying AMR in *N.* *gonorrhoeae* [36,37]. According to SRM analysis of the i19.05 and NG05 strains in comparison with n01.08, BamA was constantly less abundant in the mutant strains, while SurA, together with other chaperones taken into consideration (Skp and FkpA), was detected in a significantly higher amount. Skp and FkpA along with SurA are the three major periplasmic chaperones of Gram-negative bacteria that escort OMPs during translocation from the inner to the outer membrane. Skp is a chaperone of the “holdases” family, which prevents substrate aggregation through binding in its cavity, but without directly assisting folding [38,39]. FkpA is a chaperone that actively binds with OMP and prevents its aggregation under heat shock conditions [40]. Although the exact contribution of each chaperone to OMP biogenesis is still controversial, we assume that the increase in the abundance of the chaperones Skp and FkpA might have been a consequence of a change in the functioning of the mutant proteins SurA and BamA.

The results of LC-MS/MS analysis of NG05 and n01.08 strains demonstrated that the replacement of three genes induces changes in the representation of 10% of the proteome. COG analysis and GO function annotation revealed that proteins of extracellular structures and cell motility categories, which were represented by pilin proteins, were upregulated. The increased amount of CE proteins probably leads to the activation of ribosomes and the overabundance of secretion proteins. Besides the pilins, the proteins responsible for transport and binding to iron, copper, and manganese ions were increased. It is not yet clear whether an increase in the amount of transport and pilin proteins is associated with a decrease in the level of drug sensitivity of the bacteria, or is a consequence of changes in the quantity and quality of chaperones. On the contrary, during SurA deprivation in *Pseudomonas aeruginosa*, many proteins involved in the acquisition and transport of iron were completely or almost completely absent [19].

Moreover, an increased abundance of outer-membrane lipoprotein LolB (NGO0439) was detected in the NG05 strain. This protein is not a determinant of drug resistance but is involved in trafficking to the outer membrane of lipoproteins such as members of the efflux pumps (MtrE) and BAM-system (BamB and BamE) [41,42,43]. Moreover, in the absence of the LolB protein, *E. coli* was more sensitive to a range of antibiotics [44].

Under the pressure of antibiotics, the differences in the proteomic profile of NG05 were more considerable compared to n01.08. The most important change was the increased amount of the MacAB-Tolc efflux pumps. We also observed the same changes in the representation of MacAB-TolC efflux pumps in the original i19.05 strain. In *N. gonorrhoeae*, MacAB is involved in the active efflux of macrolides, and its loss has been linked to increased susceptibility to penicillin G and ESCs [45,46]. In a recent study [47], it was discovered that MacAB upregulation contributes to tetracycline resistance development in *Klebsiella pneumonia*. Moreover, we should not exclude that a change in the representation of periplasmic chaperones and substitutions in BamA and SurA may lead to a modification of the MacAB efflux pump.

We conducted an ethidium bromide uptake assay to test the effect of the upregulated MacA and MacB proteins on the efflux level. It was shown that the accumulation rate of the transformant strain NG05 was significantly lower than that of the recipient n01.08 and NG01. However, with the addition of PAβN, which is a well-studied broad-spectrum efflux pump inhibitor, the difference between strains disappeared. Furthermore, in the presence of the inhibitor, the MICs of tetracycline and clarithromycin decreased to equal values among strains under study. Taken together, it is suggested that the mechanism of drug resistance in NG05 is associated with efflux pumps. Efflux analysis for the *penA* single mutant NG01 also showed that this mutation did not affect the efflux.

## 5. Conclusions

The bacterial mechanisms of drug resistance are very diverse. In addition to specific mechanisms associated with the degradation of the antibiotic or a decrease in its target affinity, nonspecific ways of protection make a significant contribution. First, it is a modulation of cell membrane permeability and efflux as well as an inhibition of oxidative stress. Here, we described an unusual mechanism of gonococcus drug resistance acquisition caused by the inadequate functioning of mutant forms of SurA periplasmic chaperone and its defendant protein BamA. The replacement of these genes in the pan-susceptible strain with mutant variants led to changes in the abundance of chaperones, upregulation of the efflux pump in the presence of antibiotics, an increase in efflux, and a decrease in antimicrobial sensitivity. To confirm the causality of our substitutions in reduced susceptibility, additional lab-based efforts such as gene inactivation and replacement technology will be necessary.

## Figures and Tables

**Table 1:** MIC of antibiotics for the *N. gonorrhoeae* strains studied.

Strains	MIC Ampicillin, mg/L		MIC Tetracycline, mg/L		MIC Clarithromycin, mg/L	
	Control	PAβN *	Control	PAβN	Control	PAβN
i19.05	0.5 ± 0.16	0.12 ± 0.01	0.5 ± 0.16	0.19 ± 0.04	0.75 ± 0.16	0.19 ± 0.04
NG05 (*penA^mut^, bamA^mut^, surA^mut^*)	0.25 ± 0.06	0.12 ± 0.01	0.38 ± 0.06	0.12 ± 0.01	0.5 ± 0.16	0.19 ± 0.04
NG01 (*penA^mut^*)	0.12 ± 0.01	0.06 ± 0.02	0.25 ± 0.06	0.12 ± 0.01	0.25 ± 0.06	0.19 ± 0.04
n01.08	0.06 ± 0.02	0.03 ± 0.01	0.25 ± 0.06	0.12 ± 0.01	0.25 ± 0.06	0.19 ± 0.04

The values are representative data from three biological replicates, each performed in duplicates. * The effect of an efflux pump inhibitor on MICs is described below.

## Data Availability

The mass spectrometry proteomics data have been deposited to the ProteomeXchange Consortium (http://proteomecentral.proteomexchange.org) via the PRIDE partner repository with the dataset identifier PXD022993.

## Supplementary Materials

The following are available online at https://www.mdpi.com/article/10.3390/genes12091312/s1. Table S1: SAP in SurA and BamA of *Neisseria gonorrhoeae* i19.05 strain, Table S2: Differentially expressed proteins found in both fractions, Table S3: Functional enrichment analysis of differentially expressed proteins, Table S4: Primers selected for analysis of *N. gonorrhoeae* genes, Figure S1. Functional classification of DAPs according to COGs.
