# Supplementary material for: Substitutions in SurA and BamA Lead to Reduced Susceptibility to Broad Range Antibiotics in Gonococci
Source: Genes (Basel). 2021 Aug 25;12(9):1312. doi: 10.3390/genes12091312 (PMC8467665; doi:10.3390/genes12091312)
Supplement: Supplementary file 1 [file genes-12-01312-s001.zip › genes-1349495-supplementary/Figure S1.pdf]

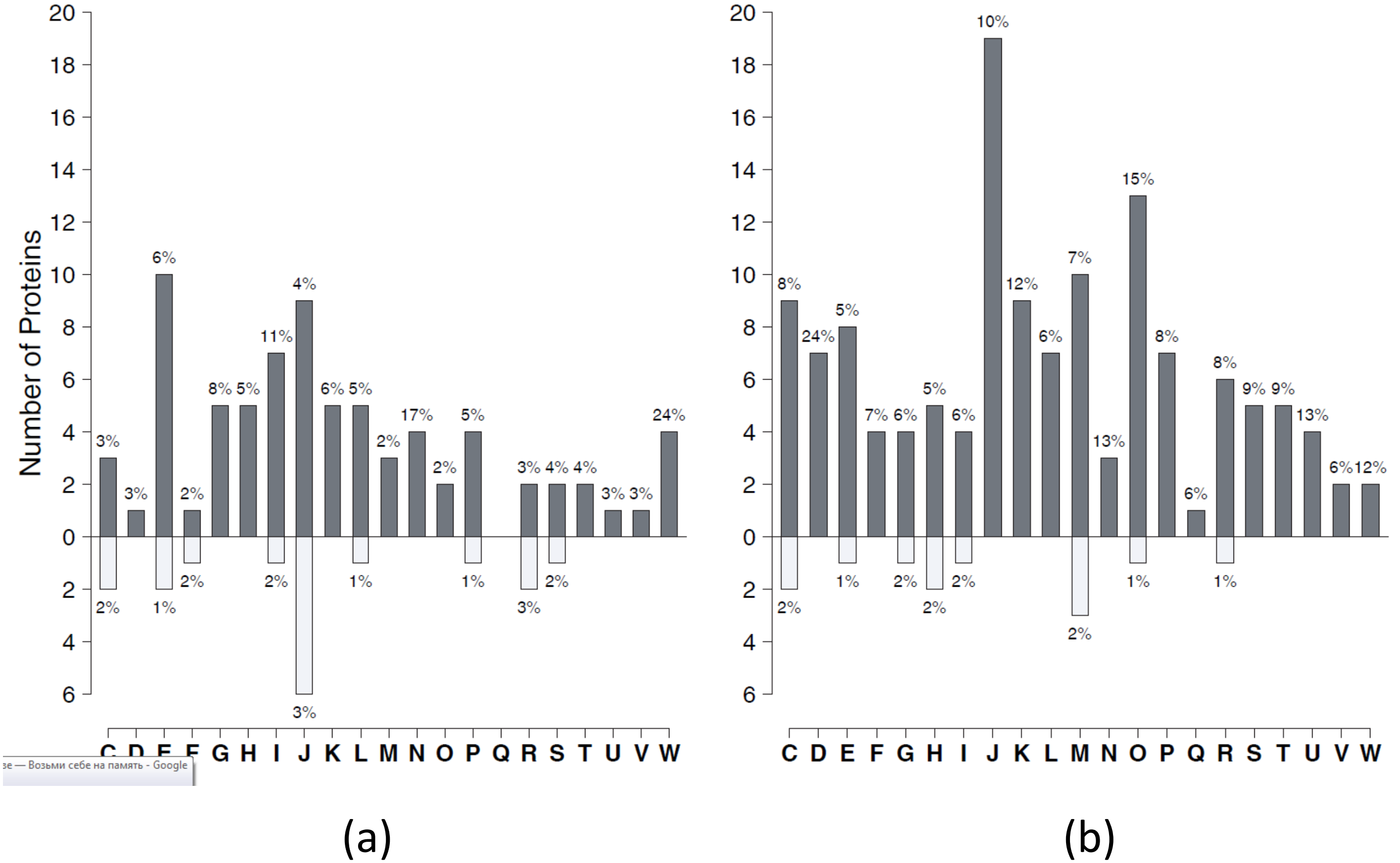


**C** Energy production and conversion
**D** Cell cycle control, cell division, chromosome partitioning
**E** Amino acid transport and metabolism
**F** Nucleotide transport and metabolism
**G** Carbohydrate transport and metabolism
**H** Coenzyme transport and metabolism
**I** Lipid transport and metabolism
**J** Translation, ribosomal structure and biogenesis
**K** Transcription
**L** Replication, recombination and repair
**M** Cell wall/membrane/envelope biogenesis
**N** Cell motility
**O** Post-translational modification, protein turnover, and chaperones
**P** Inorganic ion transport and metabolism
**Q** Secondary metabolites biosynthesis, transport, and catabolism
**R** General function prediction only
**S** Function unknown
**T** Signal transduction mechanisms
**U** Intracellular trafficking, secretion, and vesicular transport
**V** Defense mechanisms
**W** Extracellular structures

**Figure S1.** Functional classification of DAPs according to COGs. Histogram illustrating the COG functional categories and the associated protein number. (a) Proteins of cell envelope fraction; (b) proteins of the cytoplasmic fraction. The values above and below the X-axis indicate the number of increased and decreased proteins, respectively. The percentages show the number of DAPs relative to the total number of proteins in the particular COG.
